# Recurrent laryngeal nerve schwannoma adjacent to the thyroid: a case report

**DOI:** 10.1186/s13256-026-06002-1

**Published:** 2026-04-07

**Authors:** Mayu Yamauchi, Akihiro Sakai, Hiroaki Iijima, Takanobu Teramura, Aritomo Yamazaki, Ryoko Yanagiya, Toshihide Inagi, Ai Yamamoto, Yoshiyuki Ota, Hiroshi Ashida, Yurina Sato, Naoya Kobayashi, Koji Ebisumoto, Koichiro Wasano, Kenji Okami

**Affiliations:** Department of Otolaryngology, Head and Neck Surgery, Tokai University School of Medicine, 143 Shimokasuya, Isehara, Kanagawa 259-1193 Japan

**Keywords:** Recurrent laryngeal nerve, Schwannoma, Parathyroid mass, Neurorrhaphy, Schwannomatosis

## Abstract

**Background:**

Schwannomas arising from the recurrent laryngeal nerve (RLN) are exceedingly rare tumors that can present diagnostic challenges when located adjacent to the thyroid gland. This case highlights the importance of considering neurogenic tumors in the differential diagnosis of paratracheal masses.

**Case presentation:**

A 56-year-old Japanese woman was referred to our hospital after a routine chest radiograph revealed tracheal deviation. Computed tomography revealed a 4-cm mass along the left side of the trachea. Neck ultrasonography revealed a 37-mm lesion lateral to the inferior pole of the thyroid with cystic changes. Although a parathyroid tumor was considered, laboratory findings were not supportive, and fine-needle aspiration cytology was nondiagnostic. Surgical excision was performed for a definitive diagnosis and treatment. Intraoperatively, the tumor was extrathyroidal and contiguous with the RLN. The caudal end of the tumor extended close to the mediastinum, making dissection difficult. Therefore, the RLN was divided both cranially and caudally, and after tumor removal, the nerve stumps were reapproximated and sutured (neurorrhaphy). Histopathological examination confirmed the diagnosis of a schwannoma. The patient also had a history of cauda equina and posterior femoral cutaneous nerve tumors, suggestive of schwannomatosis (multiple schwannomas). At 24 months after surgery, no clinical or radiological evidence of recurrence was observed.

**Conclusions:**

RLN schwannomas are exceedingly rare; neurogenic tumors should be considered in paratrachyroid masses near the inferior pole, especially when imaging shows an extrathyroidal cystic lesion and cytology is nondiagnostic. Preoperative evaluation with ultrasonography and contrast-enhanced CT facilitates diagnosis, and surgical excision with nerve function preservation should be prioritized.

## Introduction

Schwannomas arising in the neck are common in otolaryngologic practice, and most of them originate from the vagus nerve. In contrast, schwannomas of the recurrent laryngeal nerve (RLN) are exceedingly rare, with only a few reports in the literature. Here, we describe a schwannoma adjacent to the inferior pole of the thyroid that was intraoperatively confirmed to be contiguous with the RLN, and we provide a brief review of the literature.

## Case report

**Fig. 1:**
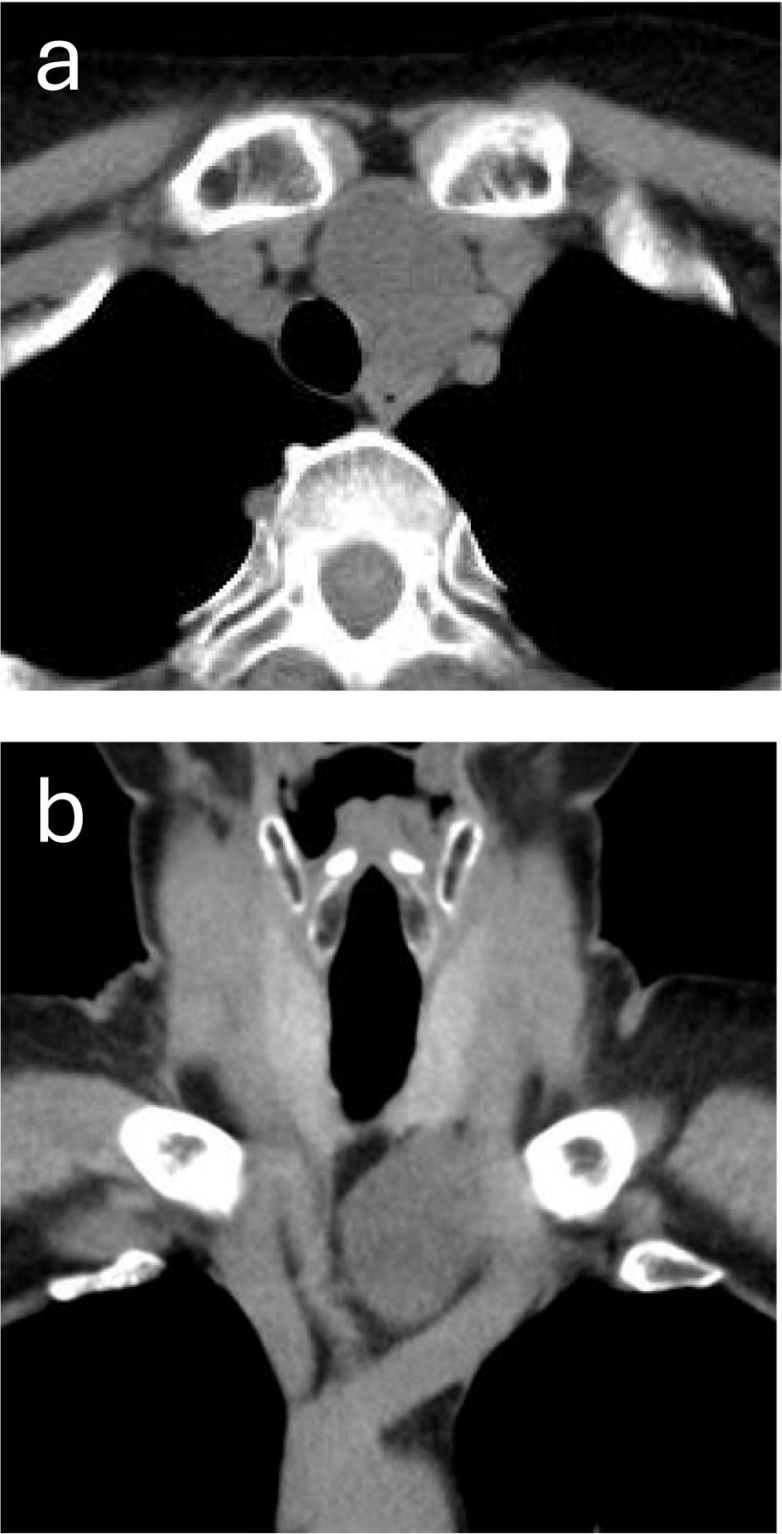
Contrast-enhanced computed tomography revealed a well-circumscribed, 4-cm mass located in the left paratracheal region (**a**, axial view). The lesion displaced and compressed the trachea medially without evidence of invasion into adjacent structures (**b**, coronal view)

**Fig. 2:**
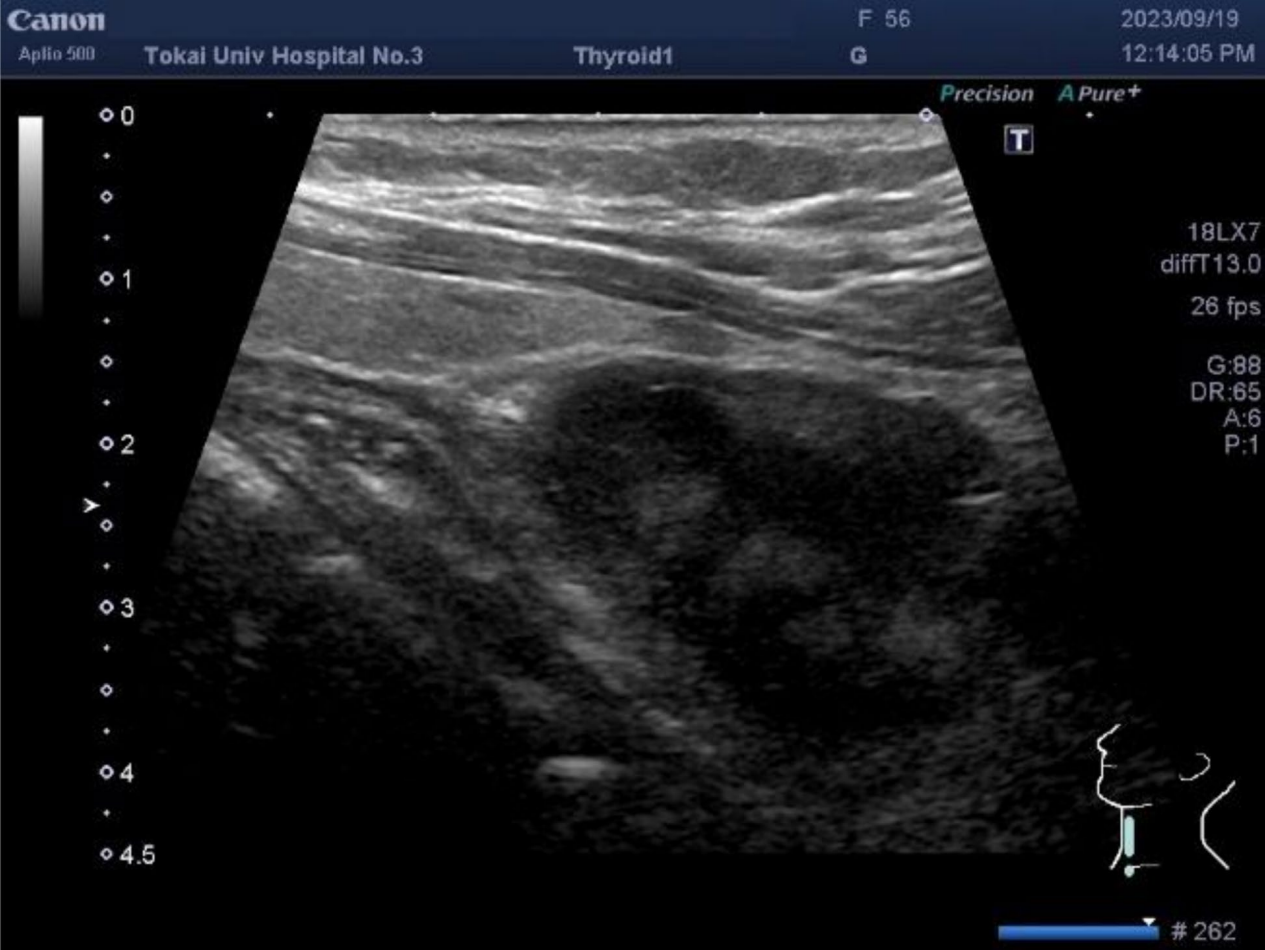
Cervical ultrasonography demonstrated a 37-mm well-defined mass located lateral to the lower pole of the thyroid gland. The lesion exhibited internal cystic degeneration

A 56-year-old Japanese woman was referred to our hospital after a routine chest radiograph revealed tracheal deviation. Computed tomography (CT) revealed a 4-cm left paratracheal mass with rightward tracheal displacement on the chest radiography (Fig. 1). Neck ultrasonography identified a 37-mm partly cystic lesion lateral to the inferior pole of the left thyroid lobe (Fig. 2). Laboratory test results, including intact parathyroid hormone, thyroid-stimulating hormone, and free thyroxine levels, were within normal limits. Fine-needle aspiration (FNA) cytology (FNAC) was nondiagnostic because of an insufficient specimen.

On presentation, no palpable cervical mass or symptoms such as hoarseness were observed, and flexible laryngoscopy showed normal vocal fold mobility.

**Fig. 3:**
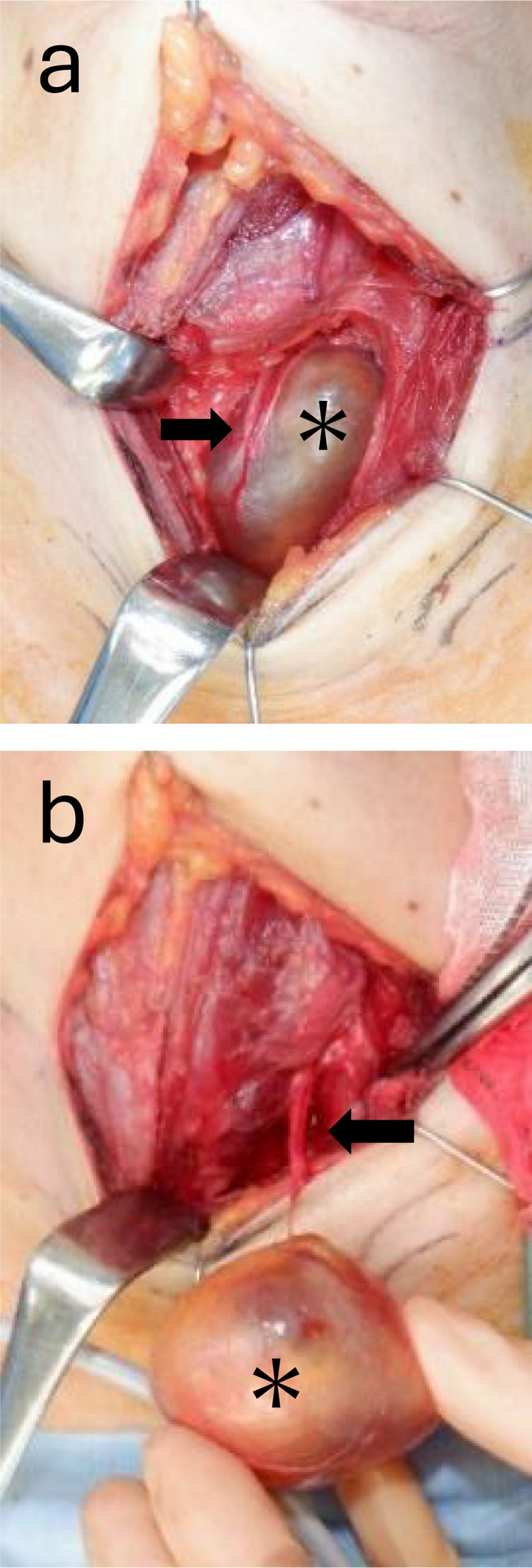
Intraoperative findings. **a** Tumor (*) was located outside the thyroid capsule and was found to be continuous with the recurrent laryngeal nerve (arrow), immediately before tumor removal. **b** Appearance during tumor removal

Given the extrathyroidal location on imaging with tracheal compression and nondiagnostic cytology, surgical management was selected. Intraoperatively, the tumor was extrathyroidal, nonadherent to the surrounding tissues, and contiguous with the RLN (Fig. 3). The caudal end extended toward the superior mediastinum, making dissection difficult. Therefore, the RLN was divided cranially and caudally; after tumor removal, the nerve stumps were reapproximated using epineurial sutures (neurorrhaphy).

The resected specimen measured 35 × 29 × 22 mm. Microscopy revealed fascicles of spindle cells, and immunohistochemistry was diffusely positive for S-100 protein, consistent with a schwannoma. Postoperatively, hoarseness due to vocal fold paralysis was observed. At the first outpatient visit, 17 days after discharge, the vocal fold position had shifted from fixed abduction to a paramedian position, and the hoarseness had resolved. The patient's medical history included a parasagittal meningioma (resected at 31 years of age), a tumor of the right posterior femoral cutaneous nerve (resected at 38 years of age), and a cauda equina tumor (resected at 47 years of age). A first-degree relative (father) also had multiple nerve tumors. These findings raised suspicion of neurofibromatosis type 2 (NF2)-related schwannomatosis, and further assessment is in progress at another institution. At 24 months after surgery, no clinical or radiological evidence of recurrence was observed.

## Discussion

Schwannomas of the neck are common benign tumors in otolaryngologic practice, most commonly arising from the vagus nerve, followed by the sympathetic chain, cervical plexus, and brachial plexus.1 By contrast, schwannomas originating from the RLN are exceedingly rare, with only a few reports available.2–4.

As schwannomas enlarge, secondary changes, such as cystic degeneration, hemorrhage, and hyalinization, are frequent, often producing internal heterogeneity on imaging.5 These features can resemble thyroid or parathyroid diseases and complicate radiologic differentiation. In our case, CT and ultrasonography revealed a mixed solid-cystic lesion extending from the lateral aspect of the inferior thyroid pole toward the tracheoesophageal groove, initially suggesting a thyroid tumor. For differential diagnosis, careful assessment should include not only intratumoral characteristics but also the continuity (or lack thereof) with the thyroid parenchyma and any relationship to an adjacent nerve trajectory. In this patient, the interface with the thyroid was partly indistinct, and an elongated, nerve-parallel morphology was not obvious; consequently, RLN schwannoma was not considered preoperatively.

Most cervical schwannomas present as asymptomatic neck masses, and neural symptoms such as hoarseness are reported in only approximately 20% of cases.6 Furthermore, the diagnostic specificity of FNA and FNAC for schwannomas is low (approximately 20%) 0.7 In our case, FNAC was nondiagnostic due to low cellularity. When an extrathyroidal mass is suspected, schwannoma should remain in the differential diagnosis irrespective of symptoms, with ultrasonography and CT used to assess contiguity with the nerve and lesion characteristics together with cytologic results.

In retrospect, the presence of multiple schwannomas and a meningioma in this patient met the diagnostic criteria for NF2-related schwannomatosis. This finding should have prompted preoperative consideration of genetic counseling and testing. After the nondiagnostic FNA result, advanced imaging modalities—particularly high-resolution MRI with dedicated attention to nerve origin—should have been pursued to better characterize the lesion. In addition, given the family history of multiple neural tumors in a first-degree relative, genetic evaluation would have been appropriate. This case underscores the importance of maintaining a systematic approach when evaluating patients with multiple neural tumors, as early recognition of hereditary tumor syndromes can inform treatment planning and enable appropriate genetic counseling for patients and their families.

Surgical excision is the mainstay of treatment for schwannomas. When the parent nerve is functionally critical, such as the RLN, vagus nerve, brachial plexus, or sympathetic chain, nerve-sparing techniques are recommended to minimize postoperative deficits. Techniques aimed at functional preservation include intracapsular (subcapsular) enucleation8 and intercapsular resection between the tumor capsule and the epineurium.9 In our case, the caudal RLN was divided, because the tumor extended toward the superior mediastinum and was difficult to mobilize; however, epineurial neurorrhaphy was performed after en bloc removal, and the patient achieved a favorable functional outcome without persistent hoarseness.

The patient also had a history of multiple schwannomas and meningiomas, and her father, a first-degree relative, had multiple nerve tumors. Although these findings do not fulfill the criteria for a definite diagnosis, they are compatible with NF2-related schwannomatosis, suggesting possible involvement. While a predilection for specific nerves in patients with multiple schwannomas has not been firmly established, an estimated 25–40% of peripheral schwannomas occur in the neck.10 Accordingly, in patients with a history of schwannoma, newly detected cervical masses should prompt consideration of cervical schwannoma in the differential diagnosis.

This case report has several limitations inherent to single-case studies. As an isolated case, the findings cannot be generalized to all patients with RLN schwannomas. Long-term follow-up data beyond 12 months would provide more robust evidence regarding the durability of functional outcomes and recurrence rates. In addition, the optimal surgical approach may vary depending on tumor size, location, and individual patient factors, which cannot be fully addressed by a single-case presentation. Furthermore, the 24-month follow-up period, while adequate for assessing benign tumor recurrence, may be insufficient for comprehensive evaluation of long-term functional outcomes and potential manifestations of genetic mosaicism. Longer-term follow-up would be valuable to assess the durability of vocal fold function and to monitor for any additional tumor development. Another significant limitation is the absence of quantitative voice assessment metrics, such as the Voice Handicap Index (VHI) score, maximum phonation time, or acoustic analysis parameters. The inclusion of standardized voice outcome measures would have provided more objective data regarding the functional impact of RLN preservation and should be considered essential in future reports of RLN schwannoma cases.

## Conclusion

Here, we report a rare case of a schwannoma arising from the extralaryngeal (cervical) segment of the RLN. Neurogenic tumors should be included in the differential diagnosis of cervical masses near the inferior thyroid pole. Preoperative evaluation with ultrasonography and contrast-enhanced CT, assessing continuity with the thyroid parenchyma, relationships with the tracheoesophageal groove and carotid sheath, and contiguity with the nerve course, facilitates the diagnosis. Surgical excision remains the standard treatment, and nerve function preservation should be prioritized. The operative technique (e.g., subcapsular [intracapsular] enucleation or intercapsular resection) should be selected based on the individual case. In patients with a history of schwannoma, a newly detected cervical mass should again prompt consideration of a cervical schwannoma.

## Data Availability

All data generated or analyzed during this study are included in this published article.
